# Nutrition Screening and Counseling in Patients With Lung Cancer in an Outpatient Setting

**DOI:** 10.6004/jadpro.2012.3.3.9

**Published:** 2012-05-01

**Authors:** Susan Snight Moreland

**Affiliations:** From The Catholic University of America, Washington, DC

Malnutrition is evident in 30% to 90% of patients with cancer at some point during their disease course (Molina, Yang, Cassivi, Schild, & Adjei, 2008; Nitenberg & Raynard, 2000; Read et al., 2005). Significant weight loss (> 10% of body weight) increases the risk of morbidity and mortality in individuals with cancer (Bozzetti, 2009; Dewys et al., 1980; Nitenberg & Raynard, 2000; Ottery, 1996). At diagnosis, at least one third of patients exhibit weight loss of > 5%, but this number is significantly higher in those with advanced disease (Ottery, 1996). Along with this weight loss, there are also changes in protein and albumin status that indicate progressive deterioration of nutritional status and propensity for cachexia (Esper & Harb, 2005). Thus, it is important to recognize those patients who are at risk for malnutrition or who are already malnourished at diagnosis.

In an outpatient private practice, a dietitian or nutritionist may not be available for nutrition counseling; thus, this important step becomes an essential component of the oncology advanced practitioner’s role. In this article, an intervention developed at a suburban outpatient practice to address the problem of malnutrition in lung cancer patients is described. A synopsis of the screening and counseling program is given.

## Initial Office Visit

At the first office visit, newly diagnosed lung cancer patients were approached to participate in the pilot study. Seven patients (four males, three females) consented to participate. One participant had stage II disease, three had stage III disease, and three had stage IV disease. Participants ranged in age from 45 to 83 years and all were either current (six patients) or former (one patient) smokers. Six of the participants had a diagnosis of non–small cell lung cancer and one had small-cell lung cancer.

During the first visit, baseline bloodwork was ordered: complete blood cell (CBC) count with differential, comprehensive metabolic panel, and C-reactive protein (CRP). The CRP was added because evidence shows that there is elevation of the acute proinflammatory humoral response in patients who are at risk for malnutrition and cachexia (J. Brown, personal communication, April 2, 2009; Esper & Harb, 2005; Nitenberg & Raynard, 2000; Slaviero, Read, Clarke, & Rivory, 2003). Each patient made an appointment for an individualized counseling session during the next week.

## Initial Individualized Nutrition Counseling Session

**Figure 1:**
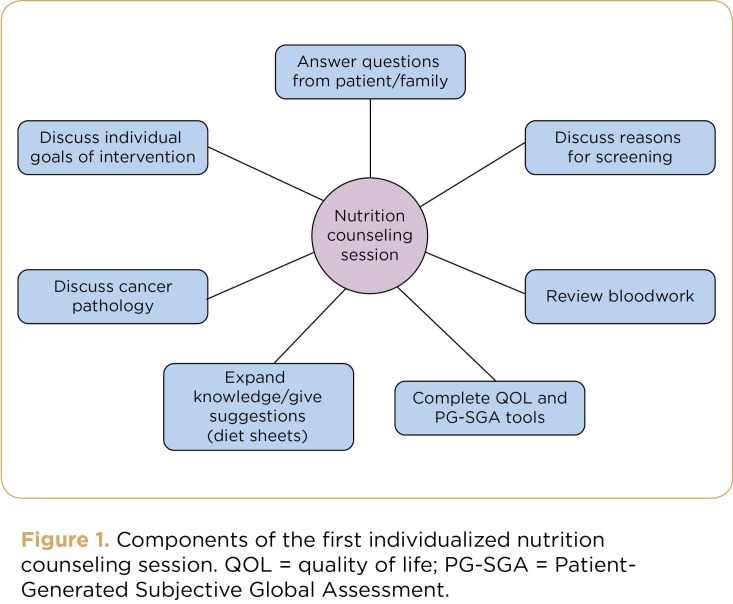
Figure 1. Components of the first individualized nutrition counseling session. QOL = quality of life; PG-SGA = Patient-Generated Subjective Global Assessment.

Each individualized nutrition counseling session with the patient and family members included an explanation of the nutrition screening, setting of individual goals for the intervention, bloodwork review, explanation and completion of required screening tools, and discussion of nutrition information. During the first session, the project goal was explained to the patient and family members as being the successful screening for nutritional deficiencies, the evaluation of any deficiencies, and the provision of counseling and support during therapy to mitigate any deficiencies. The patient and family members also shared their goals for study participation. See Figure 1 for a flow chart depicting the components of the first individualized nutrition counseling session.

The baseline bloodwork review highlighted areas of concern. In particular, the CBC and other indices that are important in evaluating for nutrition deficiencies (WBC count, hemoglobin, hematocrit, and platelet count) were explained to the patient and family members. The nurse practitioner investigator discussed albumin and its role as an indicator of nutrition status. The patient’s CRP level was noted, and its significance as it pertains to nutrition was explained. A pamphlet on nutrition information from the American Institute for Cancer Research (2007), entitled *Nutrition of the Cancer Patient: Special Population Series*, was distributed. This series of pamphlets focuses on cancer patients in active treatment and is broken down into understanding the side effects of therapy, treating them at home, and avoiding the complications in the future. Information sheets with suggestions on increasing caloric intake through shakes and protein-rich foods were distributed and reviewed. During the session, the patient completed the Scored Patient-Generated Subjective Global Assessment (PG-SGA) tool (Ottery, 1996).

The PG-SGA tool evolved from the Subjective Global Assessment, which was first used in the 1980s to determine a hospitalized patient’s nutrition status (Baker et al., 1982). In 1996, the PG-SGA tool was modified, by Faith Ottery at Fox Chase Cancer Center, to reflect the particular concerns related to oncology patients. The instrument has a 98% specificity and an 82% sensitivity. The top section is completed by the patient and/or family member with regard to weight changes, amount of food intake, symptoms caused by cancer or therapy, and current activity level. In the lung cancer study, the nurse practitioner investigator completed the remainder of the tool. The worksheet is made up of categories that score for weight loss, criteria or condition (other comorbidities), metabolic stress, physical examination, and global assessment. The investigator’s and patient’s scores are combined to obtain an overall assessment score. The patient and/or family member also completed a quality-of-life tool for the study, but this is outside the scope of this article and will not be discussed.

This counseling process was repeated 6 and 12 weeks after starting therapy and coincided with follow-up scans for determination of response to therapy. The patients and their family members were also given the nurse practitioner investigator’s contact information for any questions that might arise between counseling sessions.

## Results

Based on analysis of aggregate data from phone conversations with patients and family members, results from the pilot study indicate that the patients and their family members felt that both undergoing individualized counseling and being assigned a designated contact person were beneficial.

There was a correlation between CRP level and physical well-being. Patients with a higher CRP level, indicating greater inflammation, reported lower scores for physical well-being and vice versa. The serum albumin levels ranged from 2.4 to 4.6 mg/L, with a mean of 3.657 mg/L and a standard deviation of 0.76. In all cases, serum albumin levels either remained stable or rose during the 12 weeks of the study.

Recorded patient contacts to the nurse practitioner investigator were high in the first and last intervals. There was a direct correlation between PG-SGA score, or nutrition status, and the number of telephone questions. These data indicated that even though nutrition scores did improve over time, the participants felt that having the contact person as a resource was a positive experience. Due to the patients’ acuity and the study duration, there were only seven participants, one of whom died before completing the study. Thus, it is difficult to generalize the findings from this pilot study.

## Implications for the Oncology Advanced Practitioner

Monitoring the nutrition status of oncology patients is vital to their continued well-being. Oncology advanced practitioners play a unique role, which allows them to screen and counsel these patients during their treatment. The pilot study results discussed in this article describe positive effects from an intervention conducted by an advanced practice clinician in an outpatient clinic setting. Because patients are frequently in the office for an appointment, it is an easy way to maintain contact and manage any side effects from treatment before they can cause major problems. However, further research is needed on the best way to empower these complicated patients with the information and tools necessary to sustain their nutrition during the disease process. As advanced practitioners, we need to do our part to collect this evidence and incorporate it into our practice patterns.
